# Direct Observation of Nanometer-Sized Steps of Single Myosin VI Molecules in Living Cells

**DOI:** 10.1021/acs.nanolett.5c06046

**Published:** 2026-03-09

**Authors:** Quang Quan Nguyen, Jiamin Zeng, Truong Son Bui, Kilian Roßmann, Yandong Yin, Johannes Broichhagen, H. Lee Sweeney, Hyokeun Park

**Affiliations:** † Department of Physics, 58207 The Hong Kong University of Science and Technology, Clearwater Bay, Kowloon 999077, Hong Kong SAR, China; ‡ Division of Life Science, The Hong Kong University of Science and Technology, Clearwater Bay, Kowloon 999077, Hong Kong SAR, China; § Institute of Chemical Biology, 551667 Shenzhen Bay Laboratory, Gaoke Innovation Center A2008, Guangqiao Road, Guangming District, Shenzhen 518132, Guangdong, China; ∥ Leibniz-Forschungsinstitut für Molekulare Pharmakologie (FMP), Berlin 13125, Germany; ⊥ Department of Pharmacology and Therapeutics and the Myology Institute, University of Florida College of Medicine, Gainesville, Florida 32610, United States

**Keywords:** Live-cell imaging, single-molecule biophysics, stepping mechanism, myosin VI, unconventional myosin, single particle tracking

## Abstract

Living cells undergo dynamic biological processes. For example, motor proteins transport cargos by taking nanometer-sized steps. However, it is challenging to measure nanometer-sized steps in living cells. Using cell-permeable, extremely bright, and photostable deuterium congeners of tetramethyl­(silicon)­rhodamine (SiR-d12) connected chloroalkane linker to label single HaloTag-fused myosin VI in living cells and total internal reflection fluorescence microscopy (TIRFM), we measured nanometer-sized steps of single myosin VI in living cells. The measured step size of wild-type myosin VI was larger than that predicted from its short-lever arms. Furthermore, myosin VI harboring a mutation in the ATP-binding pocket exhibited longer dwell times between steps, reduced velocity, and shorter run lengths than wild-type myosin VI, underscoring the critical role of the ATP-binding pocket in motility. Therefore, our direct measurements of nanometer-sized steps of single motor proteins in living cells provide mechanistic insights into the dynamics and biological processes of motor proteins in living cells.

## Graphical abstract

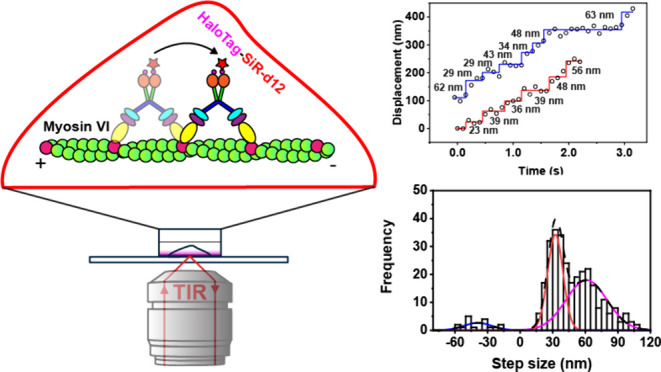

Living cells are highly dynamic and undergo continuously essential biological processes including the synthesis, transport and degradation of molecules, as well as the reorganization of cellular structures.^1^ These biological processes exhibit intricate dynamics, arising from the complex interplay of cellular components, which play an important role in cell survival and responses to diverse stimuli.^2^ Understanding these complex dynamics in living cells requires real-time observation of the biological processes. Particularly, quantitative real-time measurements of biological processes are essential for comprehensive understanding of fundamental mechanisms of living cells.^3,4^ However, such measurements in living cells are hindered by technical limitations including lack of tools in specific labeling in living cells and high background signals caused by autofluorescence inside cells.^5–7^ Recently, there has been progress in imaging techniques.^8–11^ The cell-permeable HaloTag ligand has been shown to cross the plasma membrane and label successfully specific proteins in living cells.^8^ Moreover, MINFLUX microscopy demonstrated nanometer-scale spatial resolution at microsecond temporal resolution, allowing real-time tracking of single molecules.^12–14^ However, nanometer-sized measurements of biological processes, including the stepping dynamics of molecular motor proteins, remain a significant challenge.

Molecular motor proteins, including myosin, kinesin, and dynein, play a crucial role in biological processes including intracellular transport by driving unidirectional motion along the cytoskeleton with energy from ATP hydrolysis. Myosin moves along actin filaments, whereas kinesin and dynein move along microtubules.^15^ The myosin superfamily facilitates many cellular processes including intracellular transport, muscle contraction, and cell movement.^16–21^ Among the myosin superfamily, myosin VI is the only myosin to move toward the pointed (or minus (−)) end of actin filaments,^22,23^ which enables its critical role in endocytosis.^24,25^ Monomeric myosin VI contains two calmodulin (CaM) binding domains and constitutes a shorter putative lever arm compared with myosin V.^26–29^ Dimerized myosin VI was reported to take processive motion along actin filaments.^30^ *In vitro* motility assays of single myosin VI reported that the step size of myosin VI was around 30 nm, which is larger than the predicted step sizes based on its short-lever arms.^22,30–32^ Moreover, myosin VI has broad step size distribution,^22,30–32^ whereas other processive motor proteins such as myosin V have a narrow step size distribution.^31,34^ Myosin VI was reported to take steps via a hand-over-hand mechanism,^30,32,35^ but more recent *in vitro* motility assays suggested that myosin VI switches between hand-over-hand stepping and a small inchworm-like stepping mechanism.^36^ These conflicting observations underscore a fundamental unresolved question: how does myosin VI take steps in living cells? Addressing this question is critical because the stepping behavior of myosin VI directly governs its roles in intracellular cargo transport and other essential cellular processes.

Although *in vitro* motility assays have provided valuable insights into the stepping mechanisms of myosin VI, their findings may not fully reflect the real behavior of myosin VI in living cells. Moreover, the intracellular environment is more complex than *in vitro* systems, featuring molecular crowding, a dynamic cytoskeletal network, and a multitude of regulatory proteins that can modulate motor activity, processivity, and cargo interactions. Consequently, it remains unclear whether the motility characteristics of myosin VI observed *in vitro*, including step size, run length, and velocity, are recapitulated in living cells. Thus, direct measurements of nanometer-sized steps of myosin VI in living cells are essential for understanding the accurate motility mechanisms of myosin VI under physiological conditions. However, the direct measurement of nanometer-sized steps of myosin VI in living cells remains challenging due to several technical difficulties including lack of specific labeling methods of myosin VI in living cells and high background from cellular autofluorescence, which reduces signal-to-noise ratios in live-cell imaging.^6–8,37,38^

To observe the nanometer-sized steps of myosin VI in living cells, we used recently developed bright deuterium congeners of tetramethyl­(silicon)­rhodamine (SiR-d12), which is a cell-permeable fluorogenic far-red dye, characterized to be extremely bright and very photostable.^39^ SiR-d12 with a chloroalkane (CA) linker handle on their 6-carboxy position (CA-SiR-d12) can pass through the plasma membrane and bind to Asp106 in HaloTag in proteins inside living cells through nucleophilic substitution, displacing chloride as the leaving group (Figure 1A).^39^ To specifically label myosin VI in living cells, HaloTag was added to the C-terminus of full-length myosin VI (Figure 1B). We transfected fibroblasts derived from Snell’s waltzer mice (*sv/sv* mice) lacking endogenous myosin VI^40–42^ with cDNA construct encoding full-length myosin VI fused to a HaloTag in the C-terminus using Lipofectamine transfection. Upon incubation, CA-SiR-d12 will enter cells and bind to HaloTag in full-length myosin VI inside transfected *sv/sv* fibroblasts (Figure 1C). Following thorough washing to remove unbound CA-SiR-d12, we observed numerous fluorescent puncta corresponding to SiR-d12-labeled myosin VI inside living fibroblasts. To confirm that CA-SiR-d12 specifically binds to the HaloTag fused to myosin VI, we expressed GFP-myosin VI-HaloTag in *sv/sv* fibroblasts and applied the same labeling protocol. We observed robust colocalization between GFP and SiR-d12 (Figure 1D and Figure S1), indicating that CA-SiR-d12 specifically labels HaloTag-myosin VI in living cells.

**Figure 1:**
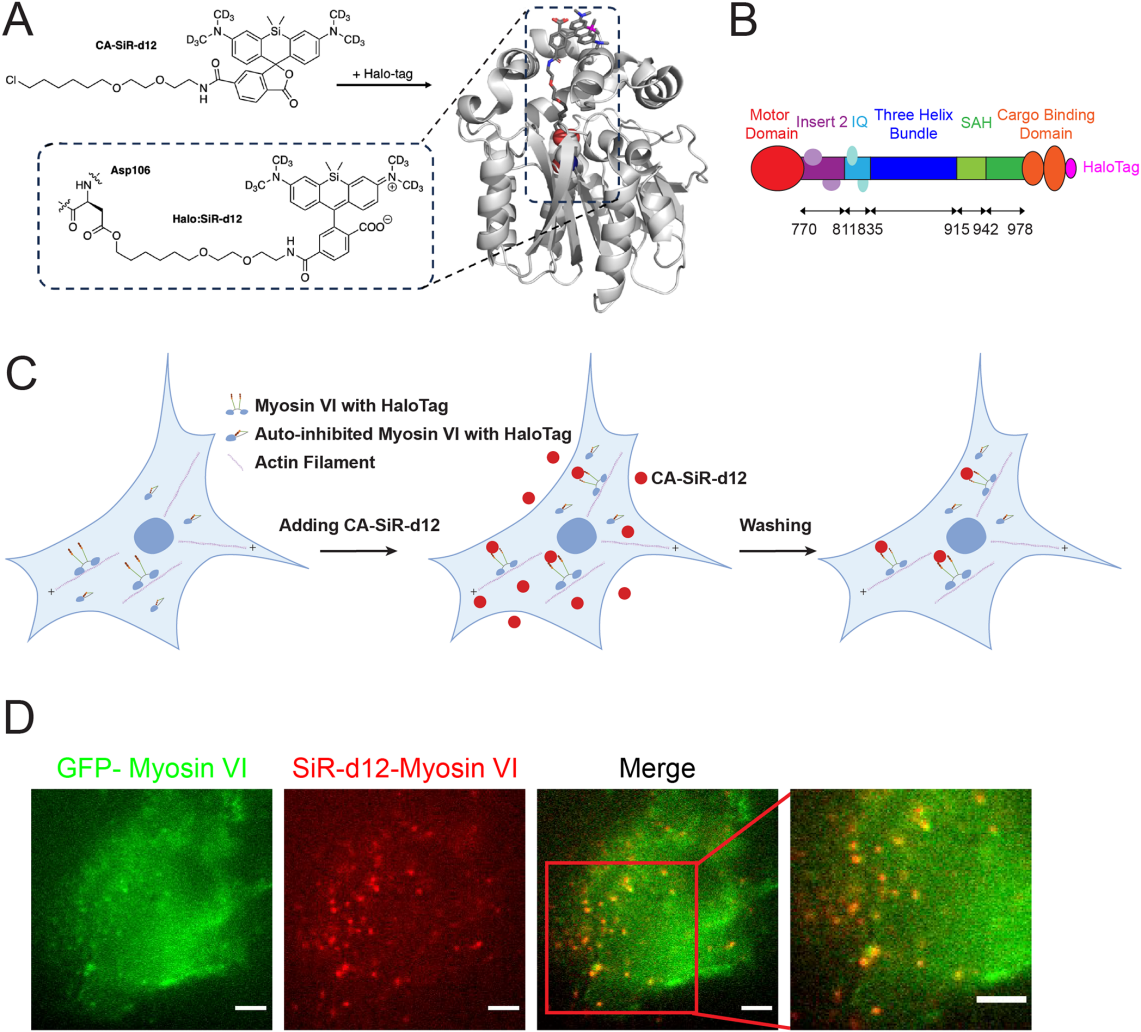
**Labeling HaloTag-fused myosin VI in living cells with CA-SiR-d12.** (A) Chemical structure of deuterium (d12) congeners of tetramethyl­(silicon)­rhodamine (SiR-d12) with a chloroalkane (CA) linker handle on the 6-carboxy position (CA-SiR-d12) and its binding to HaloTag. CA-SiR-d12 binds to Asp106 in HaloTag through nucleophilic substitution, displacing chloride as the leaving group. HaloTag is shown in gray color based on the published X-ray crystal structure (pdb: 6u32) for TMR-bound HaloTag^66^ and modified in PyMOL. (B) Schematic domains of HaloTag-fused full-length myosin VI. Following the N-terminal motor domain, myosin VI contains a distinctive insert (Insert 2), an IQ motif, a three-helix bundle, and a stable single α helix (SAH) domain and the cargo-binding domain. The C-terminus of full-length myosin VI was engineered to incorporate a HaloTag for site-specific labeling with CA-SiR-d12. (C) Schematic of labeling HaloTag-fused myosin VI with CA-SiR-d12. Cells were transiently transfected with a plasmid encoding myosin VI containing HaloTag in the C-terminus. Following transfection, cells were incubated with CA-SiR-d12, which binds to HaloTag-fused myosin VI. Unbound CA-SiR-d12 was subsequently removed by thorough washing. (D) Representative image of GFP-myosin VI (green) and myosin VI-SiR-d12 (red). The merged image showed the colocalization between GFP and SiR-d12 signals (yellow), providing evidence of specific binding of SiR-d12 to HaloTag-fused myosin VI. A scale bar represents 5 μm.

To assess potential nonspecific binding of CA-SiR-d12 in living cells, we incubated CA-SiR-d12 in *sv/sv* fibroblasts that do not express HaloTag-fused myosin VI and imaged these cells under the same conditions. No detectable fluorescence signal of SiR-d12 was observed in the absence of HaloTag-myosin VI, whereas robust fluorescence of SiR-d12 was detected in *sv/sv* cells expressing HaloTag-myosin VI (Figure 2A). Quantitative analyses revealed that 0% of cells lacking HaloTag-fused myosin VI exhibited the fluorescence signal of SiR-d12 (*N* = 3 experiments), whereas 93.3 ± 6.7% of cells expressing HaloTag-myosin VI exhibited the fluorescence signal of SiR-d12 (*N* = 3) (Figure 2B). These results confirm that CA-SiR-d12 specifically labels HaloTag-fused myosin VI in living cells without nonspecific binding.

**Figure 2:**
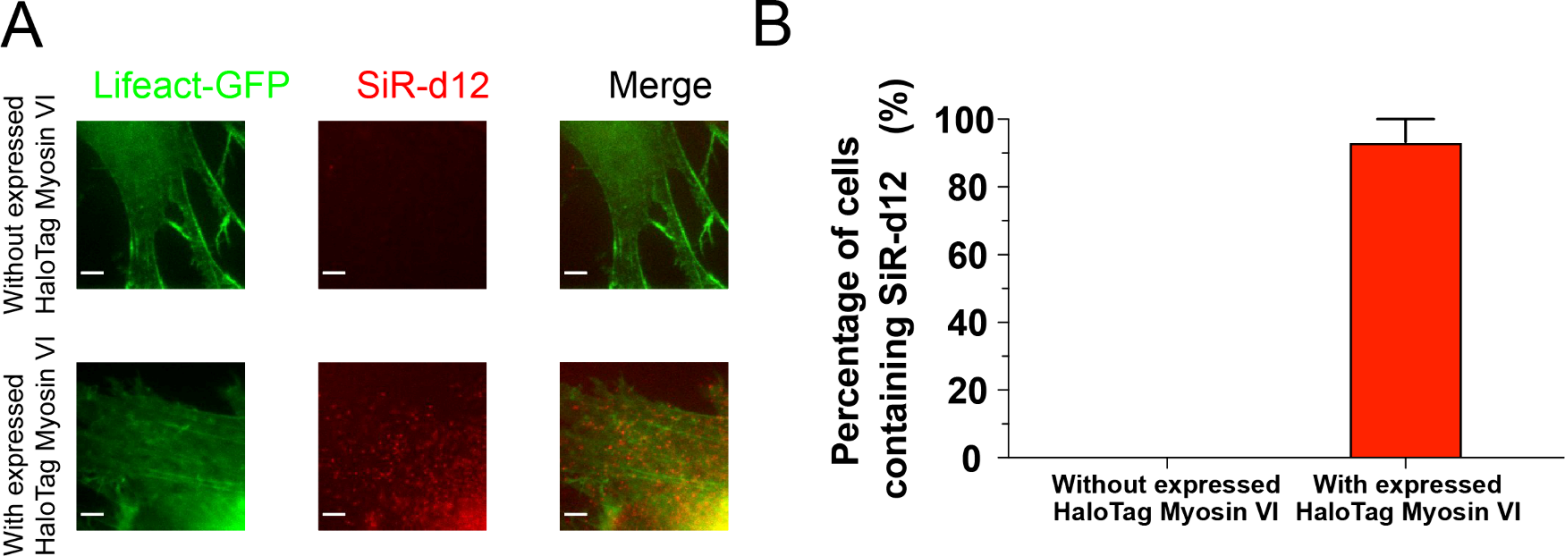
**No nonspecific binding of CA-SiR-d12 in living cells.** (A) Representative fluorescence images of cells transfected with Lifeact-GFP alone (top) and with Lifeact-GFP along with HaloTag-fused myosin VI (bottom) after incubation of CA-SiR-d12. Signals from Lifeact-GFP (green) and SiR-d12 (red) were acquired simultaneously. No SiR-d12 signal was detected without transfection of HaloTag-fused myosin VI, whereas numerous fluorescence puncta of SiR-d12 were observed in cells transfected with HaloTag-fused myosin VI. A scale bar represents 5 μm. (B) Percentage of cells exhibiting fluorescence signals of SiR-d12 without and with expressed HaloTag-fused myosin VI. Three independent experiments were performed; 93 ± 6.7 (SEM)% of cells transfected with HaloTag-fused myosin VI showed fluorescence signals of SiR-d12, whereas 0% of cells transfected with Lifeact-GFP alone showed fluorescence signals of SiR-d12, indicating that there was no nonspecific binding of CA-SiR-d12 in living cells.

The specific labeling of HaloTag-fused myosin VI in living cells with CA-SiR-d12 allowed for measurement of the step sizes of myosin VI in myosin VI-deficient *sv/sv* fibroblasts. First, we quantified the localization precision of CA-SiRd12 in fixed *sv/sv* fibroblasts using total internal reflection fluorescence microscopy (TIRFM) to decrease background. The localization precision, defined as the standard deviation (σ), was 3.6 nm along the x-axis and 4.4 nm along *y*-axis (Figure S2), which enables us to detect nanometer-sized steps of single myosin VI motors. We observed numerous fluorescence puncta of SiR-d12-labeled myosin VI inside the plasma membrane of living cells by using TIRFM (Figure 3A). We analyzed SiR-d12-labeled myosin VI showing unidirectional motion in the cytosol. SiR-d12-labeled myosin VI shown in the inset of Figure 3A moved unidirectionally around 1.5 μm in living cells before one-step photobleaching (Figure 3B). The one-step photobleaching indicates that one SiR-d12 molecule bound this myosin VI protein. To localize the centroid of one SiR-d12 with accuracy of nanometers, the original fluorescence signals were fitted into a two-dimensional Gaussian function using fluorescence imaging with one-nanometer accuracy (FIONA)^43,44^ (see the details in the Supporting Information). Figure 3C shows a representative trace of SiR-d12-labeled myosin VI in living cells, and the trace showed discrete stepping of single SiR-d12-labeled myosin VI, confirming that our method can directly resolve nanometer-sized steps of individual motors in living cells.

**Figure 3:**
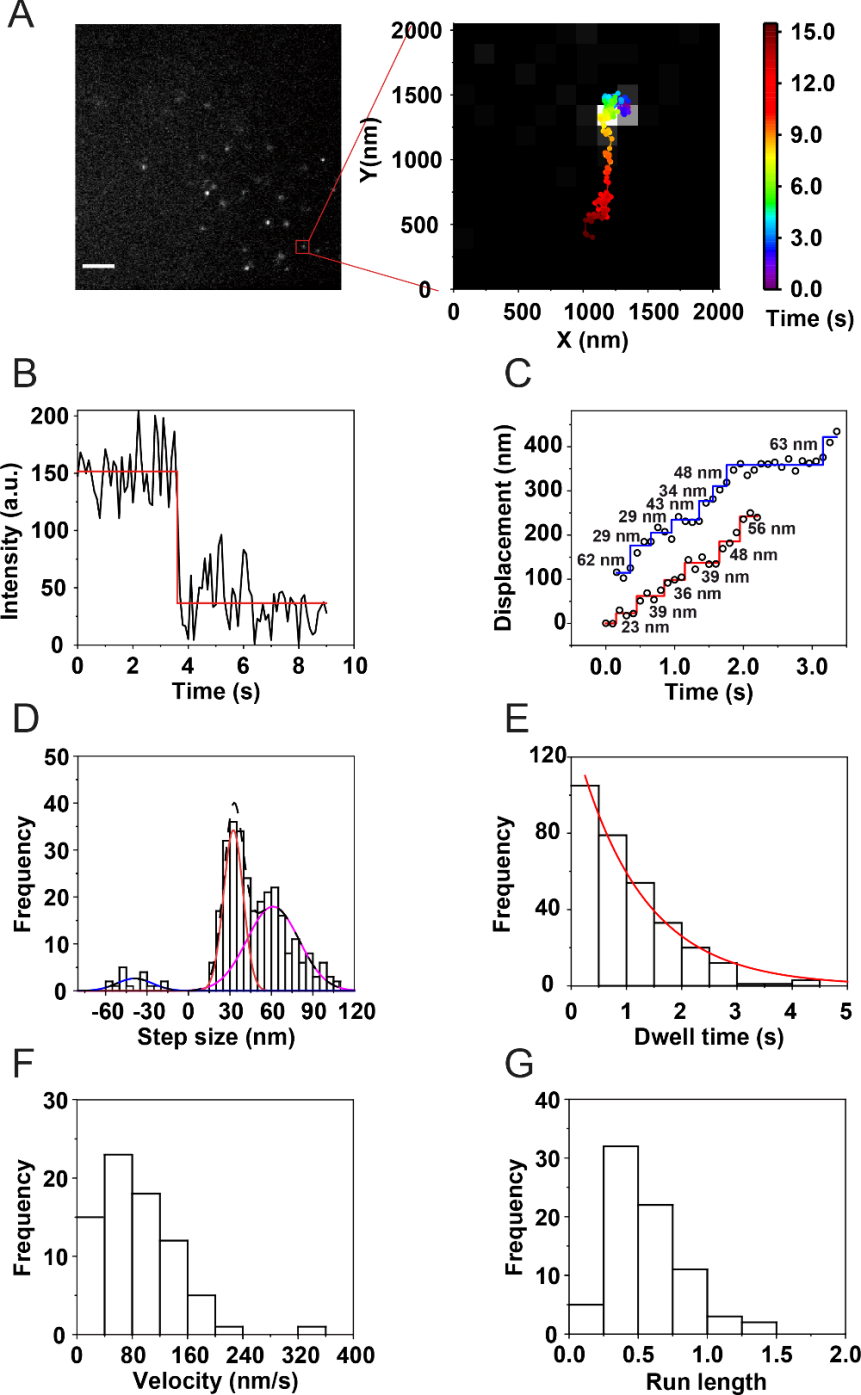
**Measurement of nanometer-sized steps of single wild-type myosin VI in living cells.** (A) Representative fluorescence image of SiR-d12-labeled myosin VI in a living cell taken at 6.1 s after the initiation of imaging. An expanded field of view shows a SiR-d12-labeled myosin VI exhibiting unidirectional motion. A scale bar represents 5 μm. A color bar represents elapsed time. (B) Fluorescence intensity of SiR-d12-labeled myosin VI showed one-step photobleaching, indicating that this myosin VI contained one SiR-d12 molecule. (C) Representative trace of a SiR-d12-labeled myosin VI exhibiting stepwise motion. (D) Step size histogram of SiR-d12-labeled myosin VI in living cells. The histogram was fitted with a sum of three Gaussian functions, each centered at −40.0 ± 12.9 (±SD), 32.5 ± 7.1, and 61.2 ± 18.9 nm (*n* = 308 steps). (E) Dwell time histogram of SiR-d12-labeled myosin VI in living cells. The histogram was well fitted with a single exponential function (*f*(*t*) = *A* × *k* × e^–*kt*^) with *k* = 0.82/s (*n* = 308). (F) Velocity histogram of SiR-d12-labeled myosin VI exhibiting stepwise motion (*N* = 75 myosin VI motors) in living cells. The average was 90.7 ± 56.8 nm/s. (G) Run length histogram of SiR-d12-labeled myosin VI exhibiting stepwise motion (*N* = 75) in living cells. The average run length was 0.54 ± 0.25 μm.

Using the traces of single SiR-d12-labeled myosin VI in living cells, we calculated the step sizes of SiR-d12-labeled myosin VI and constructed the histogram of step sizes of SiR-d12-labeled myosin VI in living cells. The distribution of step sizes of SiR-d12-labeled myosin VI exhibited an asymmetrically broad profile (Figure 3D), similar to the previous reports from *in vitro* motility assays.^22,30–32,36^ The histogram of step sizes was well fitted to a sum of three Gaussian functions using the expectation-maximization algorithm.^45–48^ Gaussian fitting revealed three centers of step sizes of SiR-d12-labeled myosin VI in living cells at −40.0 ± 12.9 (±standard deviation (SD)), 32.5 ± 7.1, and 61.2 ± 18.9 (*n* = 308 steps) nm. The first step size of SiR-d12-labeled myosin VI represents the occasional backward steps of myosin VI, consistent with the previous reports.^22,30–32,36^ The second step size (32.5 nm) corresponds to the forward step size of myosin VI labeled with single SiR-d12 in the C-terminus. The third step size of myosin VI (61.2 nm) was nearly twice that of the forward step size (32.5 nm), suggesting that this large step size likely results from two rapid and consecutive steps of myosin VI occurring within the exposure time of our camera (0.1 s) in living cells. Intracellular concentrations of ATP inside living cells are typically in the millimolar (mM) range,^49^ which is substantially higher than the tens of micromolar ATP concentrations commonly used in *in vitro* motility.^22,30–32,36^ This elevated ATP level in living cells may accelerate the mechanochemical cycle of myosin VI, enabling two successive steps to occur within a single imaging frame. To further characterize the kinetics of single myosin VI in living cells, we analyzed dwell times between two consecutive steps. Figure 3E shows the histogram of dwell times between consecutive steps. Fitting the dwell time histogram into an exponential decay function (*f*(*t*) = *A* × *k* × e^–*kt*^) yielded a single rate constant (*k*) of 0.82/s (*n* = 308 steps). This corresponds to an average waiting time (1/*k*) of 1.22 s per step. The average velocity of SiR-d12-labeled myosin VI showing stepwise motion was at 90.7 ± 56.8 nm/s (*N* = 75 myosin VI motors). Additionally, the average run length for SiR-d12-labeled myosin VI showing a stepping motion was 0.54 ± 0.25 μm (*N* = 75 myosin VI motors) (Figure 3F). Collectively, these results demonstrate that labeling myosin VI in living cells with SiR-d12 enables accurate quantification of key motility parameters of myosin VI in living cells, including step sizes, velocity and run length, thereby providing a robust method for dissecting the dynamics of single motor proteins in their native cellular environment.

To determine whether our direct measurements of motility parameters, including nanometer-sized steps of single motor proteins in living cells, can elucidate the motility mechanisms of myosin VI in living cells, we performed single-molecule tracking experiments of ATP-binding pocket mutant, which replaces a leucine residue at position 310 in the ATP-binding pocket in the motor domain of full-length wild-type myosin VI with a glycine (myosin VI L310G mutant) (Figure 4A) and was shown to perturb ATP-binding of myosin VI from biochemical assays.^50^ We transiently expressed the myosin VI L310G mutant containing HaloTag in the C-terminus in living *sv/sv* fibroblasts and successfully labeled the myosin VI L310G mutant with CA-SiR-d12 in living cells for tracking of single mutant myosin VI motors. We observed a reduced population of single SiR-d12-labeled myosin VI L310G mutant moving unidirectionally (12.6% (61 out of 486 myosin VI L310G mutant motors) vs 20.2% (103 of 510 wild-type myosin VI motors) under the same experimental conditions) and reduced processivity in the cytosol compared with wild-type myosin VI. These results suggest that perturbed ATP binding alters the ability of motor proteins to sustain processive and directed motility. Single myosin VI L310G mutant molecules exhibited nanometer-sized step behavior in living cells (Figure 4B). Quantitative analysis of the step sizes revealed an asymmetrically broad distribution with multiple distinct peaks (Figure 4C), resembling wild-type myosin VI. The histogram of step sizes was well fitted to a sum of three Gaussian functions centered at −37.7 ± 5.9 (±SD), 37.3 ± 10.1, and 72.4 ± 22.8 nm (*n* = 236 steps). We further analyzed dwell times between two consecutive steps of myosin VI L310G mutant in living cells. The histogram of dwell times was well fitted with an exponential decay function (*f*(*t*) = *A* × *k* × e^–*kt*^) with a single rate constant (*k*) of 0.53/s (*n* = 236) (Figure 4D), corresponding to an average waiting time (1/*k*) of 1.90 s per step. The rate constant of the myosin VI L310G mutant in living cells was markedly reduced compared with full-length myosin VI, indicating that the mutation in L301 slows the stepping kinetics of myosin VI by perturbing ATP binding in the ATP-binding pocket. The alteration in ATP binding resulted in prolonged dwell times between successive steps, leading to a more than 50% reduction in the velocity of myosin VI L310G mutant (38.3 ± 20.4 nm/s (*N* = 33 motors)) (Figure 4E). Furthermore, the mutation decreased the run length of the myosin VI L310G mutant (0.37 ± 0.11 μm (*N* = 33)) (Figure 4F) by more than 30%. These results underscore the critical role of the motor domain—particularly residue L310—in the mechanochemical cycle and motility of myosin VI. Taken together, labeling motor proteins in living cells with SiR-d12 and measuring nanometer-sized steps in living cells reveals how a single-point mutation disrupts the dynamics of the motor proteins in living cells, offering mechanistic insights into the motility of motor proteins.

**Figure 4:**
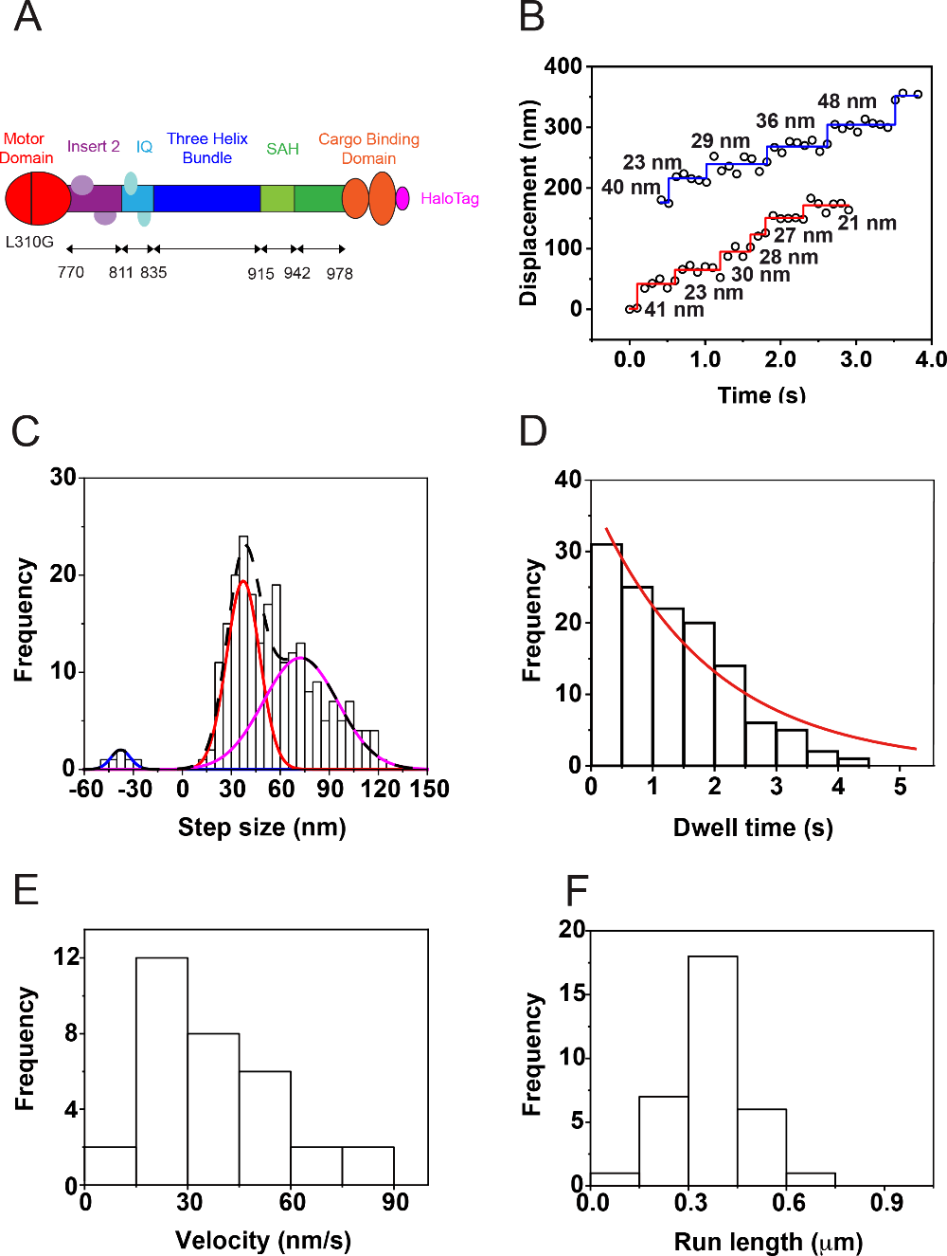
**Measurement of nanometer-sized steps of single myosin VI L310G mutant in living cells.** (A) Schematic of HaloTag-fused myosin VI L310G mutant. This myosin VI mutant harbors a leucine-to-glycine substitution at position 310 within the motor domain of the wild-type full-length myosin VI. Following the N-terminal motor domain, myosin VI L310G mutant contains a distinctive insert (Insert 2), an IQ motif, a three-helix bundle, a stable single α helix (SAH) domain and the cargo-binding domain containing HaloTag to label mutant myosin VI with CA-SiR-d12. (B) Representative trace of single SiR-d12-labeled myosin VI L310G mutant exhibiting stepwise motion in living cells. (C) Step size histogram of single SiR-d12-labeled myosin VI L310G mutant in living cells. The histogram was fitted with a sum of three Gaussian functions, each centered at −37.7 ± 5.9 (±SD), 37.3 ± 10.1 and 72.4 ± 22.8 nm (*n* = 236 steps). (D) Dwell time histogram of single SiR-d12-labeled myosin VI L310G mutant. The histogram was well fitted with a single exponential distribution function (*f*(*t*) = *A* × *k* × e^–*kt*^) with *k* = 0.53/s (*n* = 236). (E) Velocity histogram of myosin VI L310G mutant exhibiting stepwise motion in living cells. The average velocity was 38.3 ± 20.4 nm/s (*N* = 33 myosin VI L310G mutant motors). (F) Run length histogram of myosin VI L310G mutant exhibiting stepwise motion in living cells. The average was 0.37 ± 0.11 μm (*N* = 33).

*In vitro* motility assays have long been used in elucidating the stepping mechanisms of motor proteins. Despite their utility, *in vitro* motility assays face several inherent limitations. First, *in vitro* motility assays require the biochemical purification and *in vitro* reconstitution of proteins, which is a labor-intensive process that may risk perturbing their native conformation or functional integrity. Moreover, the immobilization of cytoskeletal filaments or motor proteins onto glass surfaces may impose steric hindrance or nonphysiological constraints that impede motility or alter dynamics of motor proteins. Furthermore, the artificial buffer conditions and substrate configurations used *in vitro* often differ substantially from the complex, crowded, and regulated intracellular environment. Consequently, findings from *in vitro* studies may not fully reflect the genuine biophysical behavior and coordination of motor proteins in living cells. Our method addressed these limitations by labeling HaloTag-fused motor proteins in living cells with far-red, membrane permeable, extremely bright, and photostable CA-SiR-d12 and measuring their nanometer-sized steps of single motor proteins in living cells with high spatial accuracy and minimal perturbance.

There have been efforts to detect nanometer-sized steps of single motor proteins in living cells. Recently, MINFLUX microscopy has resolved nanometer-sized steps of kinesin motors with millisecond temporal resolution within small fields of view of living cells.^13,14^ In contrast, our approach employs membrane-permeable brighter fluorophores under total internal reflection (TIR) illumination. This configuration offers broader accessibility and operational simplicity relative to the specialized instrumentation inherent to MINFLUX. Furthermore, wide-field imaging (∼80 × 80 μm^2^ on standard setups) facilitates the simultaneous tracking of numerous motor proteins. While our temporal resolution is lower, our method is well-suited for characterizing myosin motors, which exhibit larger step sizes and slower kinetics compared to kinesin motors taking the rapid 8 nm steps.

Our precise measurement of nanometer-sized steps of wild-type myosin VI in living cells revealed that myosin VI takes steps of approximately 30 nm under physiological conditions. This observed step size supports a model featuring an extended lever arm within the cellular environment (see panel A in Figure S3). Given that monomeric myosin VI contains only two CaM binding domains,^26^ structural models predicted a step size of ∼11 nm (see two possible models in Figure S3B,C).^29,51^ However, the larger step size observed in this report (32.5 nm) indicates that the lever arm of myosin VI extends within living cells. This extension of the lever arm likely enhances processivity of myosin VI along actin filaments, thereby facilitating intercellular functions of myosin VI. If 11 nm steps occur through models depicted in Figure S3B or C, these inchworm-like steps might be transient because the converter domain of the lead stepping head does not undergo rearrangement before the head contacts and binds to actin filaments in the inchworm-like step.

Furthermore, our analysis of nanometer-sized steps of myosin VI L310G mutant, which harbors a point mutation in the ATP-binding pocket, revealed that this mutant myosin VI exhibited longer dwell times between consecutive steps, resulting in reduced velocity and run length, consistent with a slowed ATPase cycle. Our observations in living cells align with prior *in vitro* findings,^52^ corroborating the functional impact of this mutation on the mechanochemical cycle of myosin VI and validating our cellular assay as a faithful reporter of motor protein behaviors.

Our method is not restricted to myosin VI but can be broadly applied to investigate the motion of other motor proteins including kinesin, dynein and other myosin proteins in living cells with high spatial accuracy owing to its versatility of SiR-d12.^53–55^ Particularly, our methods can be used to study unique stepping mechanisms of myosin X, which was reported to form antiparallel dimers^45,48,56^ and to exhibit unconventional stepping behaviors from *in vitro* motility assays.^45,47^ Thus, the direct measurement of nanometer-sized steps of myosin X in living cells will reveal the detailed stepping mechanisms under physiological conditions, which will provide important insight into the roles of myosin X in the formation of filopodia and cell migration in living cells because myosin X plays an important role in the formation of filopodia and cell migration.^21,58^

Furthermore, our method can be further extended into three-dimensional tracking in living cells by incorporating additional optics such as cylindrical lens^59,60^ and dual-focus optics planes.^63–65^ Three-dimensional measurements of nanometer-sized steps can more accurately capture the biological process in living cells because biological processes occur in three dimensions, and three-dimensional tracking eliminates projection artifacts that compromise the accuracy of two-dimensional tracking.

In summary, we report the measurements of nanometer-sized steps of single motor proteins in living cells. By labeling single HaloTag-fused myosin VI with cell-permeable, extremely bright, and photostable CA-SiR-d12 and measuring nanometer-sized steps of single myosin VI in living cells with high spatial accuracy and minimal perturbance, we uncovered detailed stepping mechanisms of single myosin VI in living cells. Furthermore, we demonstrated how a single mutation in the ATP-binding pocket in the motor domain alters the mobility of motor proteins in living cells. Thus, our direct nanometer-sized measurement of single molecules in living cells provides a powerful platform to uncover the dynamic mechanisms that underlie diverse biological processes in living cells, offering new insights into the mechanistic basis of cellular function.

## Supplementary Material



## Data Availability

The data that support the findings of this study are available from the corresponding author upon reasonable request.

## Notes

Experimental procedure about labeling myosin VI in living cells, imaging SiR-d12 labeled myosin VI in living cells and analyzing images, supplementary figures (Figures S1–S3), and additional references (PDF)
