# Supplementary material for: Direct Observation of Nanometer-Sized Steps of Single Myosin VI Molecules in Living Cells
Source: Nano Lett. 2026 Mar 9;26(10):3417–24. doi: 10.1021/acs.nanolett.5c06046 (PMC13003488; doi:10.1021/acs.nanolett.5c06046)
Supplement: Supplementary file 1 [file nl5c06046_si_001.pdf]

Supporting Information

Direct Observation of Nanometer-Sized Steps of Single Myosin VI Molecules in Living Cells

Quang Quan Nguyen[1,#], Jiamin Zeng[2,3,#], Truong Son Bui[1], Kilian Roßmann[4], Yandong Yin[3], Johannes Broichhagen[4], H. Lee Sweeney[5,*], Hyokeun Park[1,2,*]

[1]Department of Physics, The Hong Kong University of Science and Technology, Clearwater Bay, Kowloon, Hong Kong SAR, China

[2]Division of Life Science, The Hong Kong University of Science and Technology, Clearwater Bay, Kowloon, Hong Kong SAR, China

[3]Institute of Chemical Biology, Shenzhen Bay Laboratory, Gaoke Innovation Center A2008, Guangqiao Road, Guangming District, Shenzhen, 518132, Guangdong, China

[4]Leibniz-Forschungsinstitut für Molekulare Pharmakologie (FMP), 13125 Berlin, Germany

[5]Department of Pharmacology and Therapeutics and the Myology Institute, University of Florida College of Medicine, Gainesville, FL, 32610, USA

[#]These authors contributed equally to this work.

[*]To whom correspondence should be addressed. Email: Lsweeney@ufl.edu (H.L.S.) or hkpark@ust.hk (H.P.)

Materials and methods

DNA constructs and dye

Full-length myosin VI (amino acids 1-1300) was created starting from the amino acids MEDGKPVWAP and ending at the amino acids ATDMLQNLLK. Full-length myosin VI was constructed with a HaloTag at the C-terminus to link with the CA-SiR-d12 dye.[1] A linker of 5 amino acids (SGAGA) was added between the myosin VI sequence and HaloTag. Myosin VI-HaloTag mutant (myosin VI L310G mutant) was constructed from amino acids 1-1300 of myosin VI (starting from the amino acids MEDGKPVWAP and ending at the amino acids ATDMLQNLLK). This mutant construct contained 1 mutation point, which is L310G in the motor domain. SiR-d12 has excitation peak of 650 nm and an emission peak of 670 nm and was used to track the motility of single myosin VI molecules in living cells.

Cell culture

Immortalized Snell's waltzer (sv/sv) tail fibroblasts were cultured in Dulbecco's modified Eagle's medium (Gibco, 12100-061) with 10% (v/v) fetal bovine serum (Gibco, 10270-106) and 1% (v/v) penicillin–streptomycin (10,000 U mL−1, Gibco, 15114022).[2] Cells were grown in an incubator at 37 °C, 5% CO_2, and 100% humidity. Cleaned high-precision 12mm round glass coverslips (Deckglasser) were placed into 24-well plate, coated with 50 mg/mL poly-D-lysine (Sigma) and incubated for 1 hour. After incubation, the coverslips were rinsed with PBS for 3 times and dried in the incubator for 30 minutes. Immortalized Snell's waltzer (sv/sv) fibroblasts were seeded on the coverslips overnight and then transfected with HaloTag-fused myosin VI or the mutant HaloTag-fused myosin VI plasmids using lipofectamine 2000 (Invitrogen, 11668019).

Live cell staining and imaging

For live-cell imaging of transfected cells, live-cell labeling of recombinant myosin VI with HaloTag was performed with the CA-SiR-d12before imaging. Cell culture medium was exchanged for 0.2 nM CA-SiR-d12 in cell culture medium and incubated for 20 min in the incubator before changing back to culture medium and incubated for another 40 min. After incubation, the coverslip was placed into a sample chamber with FluoroBrite DMEM (Gibco, A1896701) with 10% FBS (Gibco, 10270-106) for live-cell imaging.

Total internal reflection fluorescence microscope

To acquire fluorescence signals, an inverted microscope (IX73, Olympus) with a 100X objective with a numerical aperture (NA) of 1.48 (UAPON, Olympus) was used. SiR-d12 was excited by a 640 nm laser (Coherent Inc.). The laser beam was expanded through a 15X beam expander (Edmond Optics) and was focused on the back focal plane of the objective through a lens. The lens was used to adjust the incident angle to achieve a critical angle for total internal reflection. Fluorescence signals were acquired with an EMCCD camera (iXon Ultra, Andor) with an exposure time of 100 msec via a frame -transfer mode. A dichroic mirror (ZET488/640rpc, Chroma) and an emission filter (ZET488/640m, Chroma) in a microscope were used to capture fluorescence signals. A custom-built dual-view containing a dichroic mirror (T570lpxr, Chroma) and two emission filters (ET525/50m for GFP and ET690/50m for SiR-d12, Chroma) was installed in front of a camera for simultaneous imaging.

Fixed cell experiments

To examine the myosin VI-HaloTag localization in SV/SV cells, cells with expression of myosin VI grown on the poly-L-lysine-caoted (Sigma) coverslips were treated with 10 nM CA-SiR-d12 for 40 minutes at 37℃ in 5% CO_2. The cells were fixed with 4% paraformaldehyde (PFA) on coverslips at room temperature for 15 min. Then acquire the fluorescence signals as described in live cell imaging. Data analysis is the same as live cell experiments and then analyze the displacements form the centroid of localizations of SiR-d12-myosin VI.

Data analysis

Centroids of single SiR-d12-labeled myosin VI were localized by fitting the point spread function (PSF) of SiR-d12-labeled myosin VI into a two-dimensional Gaussian using the following equation[1-2]

$$I\,(x,y) = A_0 + A_1 \text{exp}\left[-\frac{1}{2}\left(\frac{x-x_0}{\sigma_x}\right)^2 - \frac{1}{2}\left(\frac{y-y_0}{\sigma_y}\right)^2\right]$$

with A_0 is the background, A_1 is the peak intensity of the PSF, x_0 and y_0 are the centroids in the x and y direction, σ_x and σ_y are the corresponding standard deviations in the x and y direction. Custom-made IDL programs were used to localize the centroids of SiR-d12-labelled myosin VI. The trajectories were generated by plotting the centroids of SiR-d12-labeled myosin VI in every frame.

Step identification

Step identification was based on the previous paper written by Kalafut & Visscher[3].The algorithm of step identification was based on Schwarz Information Criterion (SIC), which selects a model which produces the shortest possible encoding of the data and models. Minimum dwell constraint and noise parameter were also taken into consideration to fine-tune the step fitting. Minimum dwell time was set to 200ms, and noise parameter was set as 1 as standard.

After initial metric and SIC calculations, the algorithm starts by finding the best step location that has largest chi-square reduction. The algorithm will test the step dwell time to see whether it is valid. Steps with short dwelling time (< 200ms) will be eliminated. After all steps have been determined, new SIC value will be calculated. The loop repeats as long as the new SIC is smaller than the previous SIC, which indicates better fitting model. Steps fitting visualization was plotted using MATLAB plot functions. Fitted step sizes and dwell times were then extracted and saved for interpretation. Step detections and calculations in trajectories were performed using a MATLAB program that was created in Ahmet Yildiz's lab at the University of California at Berkeley. Using the function normal MixEM of the mixtool package in R language and cross-validation technique, the histograms of step sizes were fitted with a sum of multiple Gaussian functions[4-7].

Supporting Figures

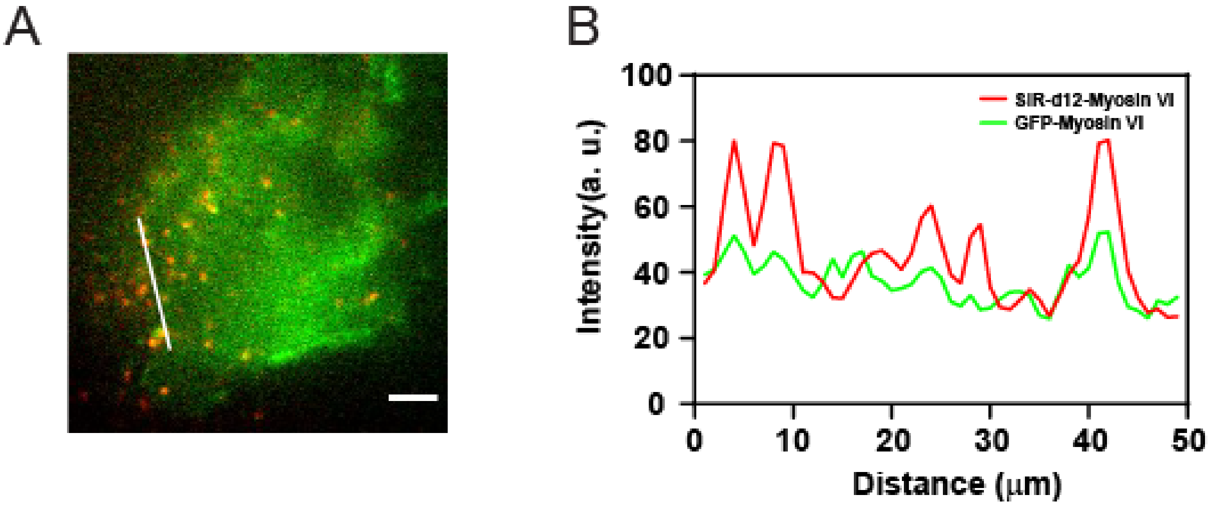

Figure S1. SiR-d12 labels HaloTag-fused myosin VI specifically
(A) Representative merged image of SiR-d12-labeled myosin VI and GFP-myosin VI in an immortalized Snell's waltzer (sv/sv) fibroblast. The merged image showed robust colocalization between SiR-d12-labeled myosin VI and GFP-myosin VI. A scale bar represents 5 μm. (B) The line scans shown in Fig. S1A reveals strong spatial colocalization between SiR-d12–labeled myosin VI and GFP-myosin VI, demonstrating that SiR-d12 labels HaloTag-fused myosin VI specifically.

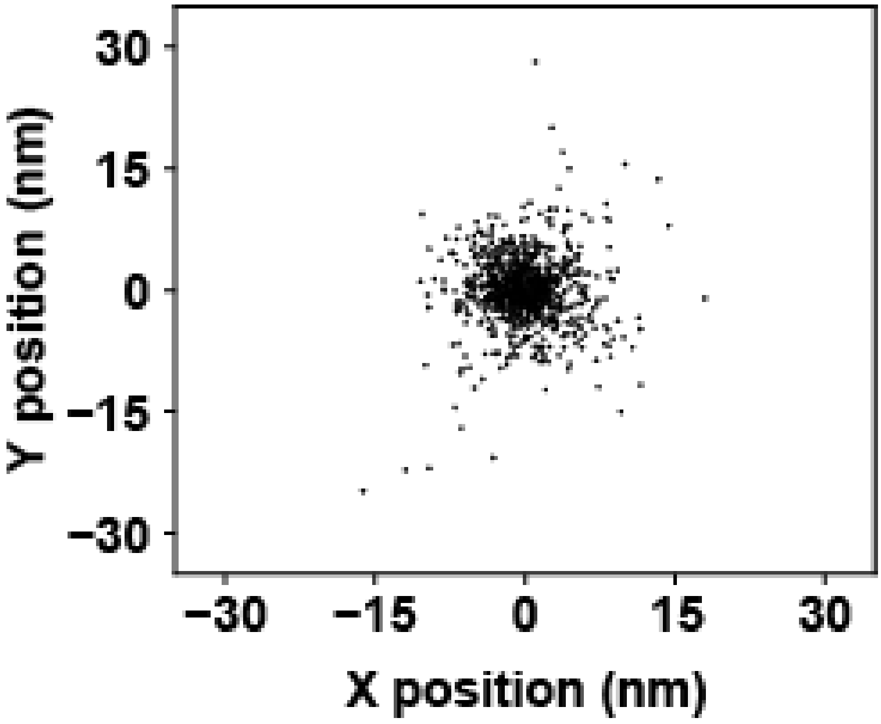

Figure S2. Localization precision of CA-SiR-d12-myosin VI.
Distribution of displacements of CA-SiR-d12-myosin VI from the centroid of their localizations in the x-y plane in the exposure time of 100 ms. 36 CA- SiR-d12-myosin VI molecules in a PFA-fixed fibroblast from sv/sv mice were used to calculate this distribution. The standard deviations (σ) in the *x*- and *y*- axes were 3.6 nm and 4.4 nm, respectively. Thus, the localization precision as the standard deviations (σ) were 3.6 nm and 4.4 nm in the *x*- and *y*- axes, respectively.

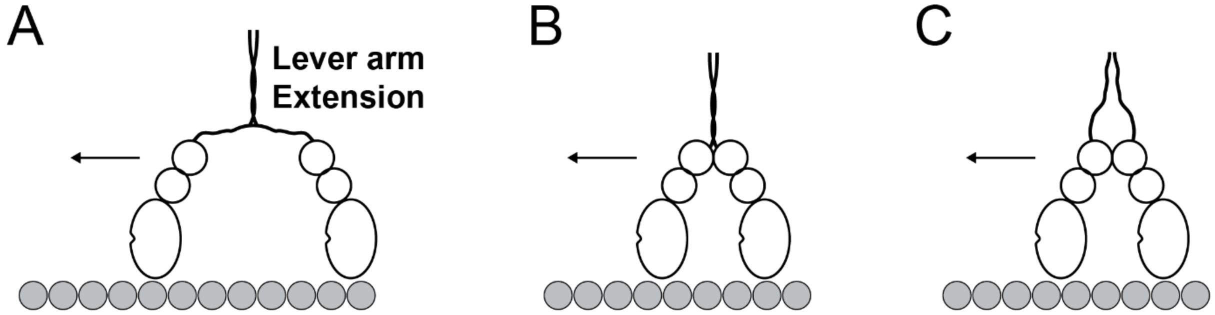

Figure S3. Stepping models of myosin VI.
(A) Model of the extended lever arm of myosin VI. The lever arm of myosin VI extends after the two calmodulin (CaM)-binding domains, which allows dimerized myosin VI molecules to take 30-36 nm steps on actin filaments. (B) Model of the folded lever arm of myosin VI. The lever arm extension remains in a folded state, which allows dimerized myosin VI takes shorter-sized steps because the reach of the stepping head is limited to 11 nm. (C) Model of the extended lever arm with restricted converter domain motion of myosin VI. The previous paper[8] demonstrate that the large lever arm swing (powerstroke) of myosin VI is made up of two structural rearrangements that happen simultaneously. There are a normal rotation of the lever arm and a rearrangement of the converter domain (region that position the lever arm and extends the lever arm out of the motor). If only the swing occurs without the converter rearrangement, then myosin VI would take a shorter step.

References

1. Park, H.; Toprak, E.; Selvin, P. R., Single-molecule fluorescence to study molecular motors. *Quarterly reviews of biophysics* **2007,** *40* (1), 87-111.

2. Park, H.; Hanson, G. T.; Duff, S. R.; Selvin, P. R., Nanometre localization of single ReAsH molecules. *Journal of microscopy* **2004,** *216* (Pt 3), 199-205.

3. Kalafut, B.; Visscher, K., An objective, model-independent method for detection of non-uniform steps in noisy signals. *Computer Physics Communications* **2008,** *179* (10), 716-723.

4. Qin, X.; Yoo, H.; Man Cheng, H. C.; Nguyen, Q. Q.; Li, J.; Liu, X.; Prunetti, L.; Chen, X.; Liu, T.; Sweeney, H. L.; Park, H., Simultaneous tracking of two motor domains reveals near simultaneous steps and stutter steps of myosin 10 on actin filament bundles. *Biochemical and biophysical research communications* **2020,** *525*, 94-99.

5. Benaglia, T.; Chauveau, D.; Hunter, D. R.; Young, D. S., mixtools: An R Package for Analyzing Mixture Models. *Journal of Statistical Software* **2009,** *32(06).*

6. Nguyen, Q. Q.; Zhou, Y.; Cheng, M. S.; Qin, X.; Cheng, H. C. M.; Liu, X.; Sweeney, H. L.; Park, H., The Antiparallel Coiled-Coil Domain Allows Multiple Forward Step Sizes of Myosin X. *The journal of physical chemistry letters* **2023**, 4914-4922.

7. Ropars, V.; Yang, Z.; Isabet, T.; Blanc, F.; Zhou, K.; Lin, T.; Liu, X.; Hissier, P.; Samazan, F.; Amigues, B.; Yang, E. D.; Park, H.; Pylypenko, O.; Cecchini, M.; Sindelar, C. V.; Sweeney, H. L.; Houdusse, A., The myosin X motor is optimized for movement on actin bundles. *Nature communications* **2016,** *7*, 12456.
8. Menetrey, J.; Llinas, P.; Mukherjea, M.; Sweeney, H. L.; Houdusse, A., The structural basis for the large powerstroke of myosin VI. *Cell* **2007,** *131* (2), 300-8.
